# Autoimmune bullous diseases in pregnancy: clinical and epidemiological characteristics and therapeutic approach^☆^^☆☆^

**DOI:** 10.1016/j.abd.2020.10.007

**Published:** 2021-07-23

**Authors:** Patrícia Penha Silveira Fagundes, Claudia Giuli Santi, Celina Wakisaka Maruta, Denise Miyamoto, Valeria Aoki

**Affiliations:** Department of Dermatology, Faculty of Medicine, Universidade de São Paulo, São Paulo, SP, Brazil

**Keywords:** Autoimmunity, Epidermolysis bullosa acquisita, Pemphigoid, bullous, Pemphigus, Pregnancy, Skin diseases, vesiculobullous

## Abstract

Autoimmune bullous dermatoses are a heterogeneous group of diseases with autoantibodies against structural skin proteins. Although the occurrence of autoimmune bullous dermatoses during pregnancy is low, this topic deserves attention, since the immunological and hormonal alterations that occur during this period can produce alterations during the expected course of these dermatoses. The authors review the several aspects of autoimmune bullous dermatoses that affect pregnant women, including the therapeutic approach during pregnancy and breastfeeding. Gestational pemphigoid, a pregnancy-specific bullous disease, was not studied in this review.

## Introduction

Autoimmune bullous diseases (AIBD) cause the appearance of bullous lesions on the skin and/or mucous membranes, resulting from the binding of autoantibodies against epidermal antigens or structures of the dermo-epidermal junction (DEJ) related to cell adhesion and are classified according to the level of epithelial cleavage.^1^ The first group, of intraepidermal bullous dermatoses, is mainly represented by pemphigus foliaceus (PF) and pemphigus vulgaris (PV); other conditions include IgA pemphigus, paraneoplastic pemphigus, and drug-induced pemphigus. There is production of autoantibodies against intercellular epidermal glycoproteins. The second group, of subepidermal bullous dermatoses, is mainly represented by bullous pemphigoid (BP) and epidermolysis bullosa acquisita (EBA), followed by less common conditions, such as linear IgA bullous dermatosis (LAD), dermatitis herpetiformis (DH), mucous membrane pemphigoid (MMP) and bullous systemic lupus erythematosus (BSLE). This group is defined by the presence of autoantibodies against the basal membrane zone (BMZ) autoantigens, leading to subepidermal cleavage.

AIBD can occur during pregnancy but are relatively rare.^2^ There are two distinct clinical situations: 1) A patient with a previously diagnosed AIBD becomes pregnant; 2) A pregnant woman with no prior history of AIBD develops an outbreak of bullous lesions that require diagnosis and management.^3^ According to this review, the first situation is the most frequent one.

Gestational pemphigoid (GP), a variant of BP, has its onset exclusively associated with pregnancy, the postpartum period, trophoblastic tumors, hydatidiform mole, or choriocarcinoma and was not included in this review.^4^

The authors aimed to carry out a review of the occurrence of AIBD during pregnancy since most publications are based on case reports.

## Immune system and pregnancy

The immune response primary purpose is to defend the host against invading pathogens and to provide tolerance to autoantigens. Thymus-derived lymphocytes, or T-lymphocytes (TL), are involved in the maintenance of this function. Thus, native TL can differentiate into TL with specificity to respond to external antigens as well as autoantigens. TL-cell maturation depends essentially on interactions with the physicochemical environment and results in the development of cells with effector and memory functions, or cells with regulatory functions. T-Helper lymphocytes play a role in immune defense by stimulating antibody synthesis, and T-regulatory lymphocytes have anti-inflammatory action and play a role in maintaining tolerance to their own cell components. Literature data indicate that, both in mice and humans, T-regulatory lymphocyte depletion is related to the development of severe autoimmune diseases.^5^

Pregnancy constitutes a specific immunological status since the maternal body must balance fetal tolerance as well as maintain its own immune system. Relevant hormonal alterations occur during pregnancy, both in progesterone and estrogen levels, as well as in cortisol, norepinephrine, and dehydroepiandrosterone concentrations. Serum progesterone and estradiol levels increase five to 10-fold throughout pregnancy, quickly returning to normal in the postpartum period.^6^ Under the influence of these hormones, profound changes occur in the immune system throughout pregnancy in order to accommodate the allogeneic fetus, which includes immunoregulatory and immunosuppressive processes. This state of immunomodulation occurs more intensely at the maternal-fetal interface, where progesterone levels are very high due to increased production of this hormone by the placenta while being more subtle in maternal peripheral blood.^7^

In the uterine region where the placenta develops (decidua), there is marked suppression of cytotoxicity, infiltration of uterine-specific natural killer (NK) cells, blocking of fetal antigens exposed to maternal lymph nodes, and accumulation of fetal-specific T-regulatory (Treg) cells. Treg cells are elevated during pregnancy, including in the maternal blood, and play a role in suppressing the local immune response through the linked suppression phenomenon. This suppression may explain the low levels of autoantibodies during pregnancy in certain autoimmune and inflammatory diseases, leading to remissions followed by reactivation in the postpartum period, such as Graves' disease and rheumatoid arthritis.^3^

**Figure 1:**
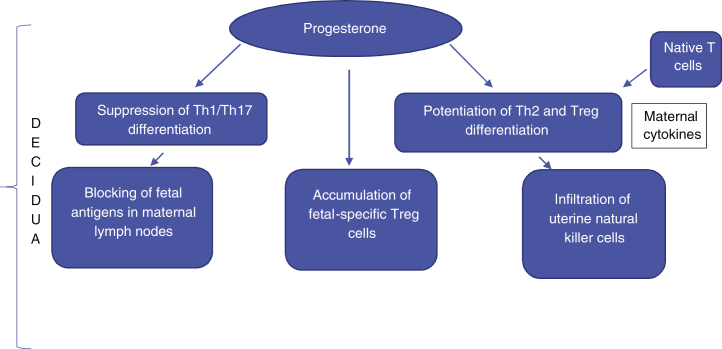
Suppression of cytotoxicity and the state of immunomodulation at the maternal-fetal interface.

There is a scarcity of data in the medical literature mentioning the important changes in the Treg cell population in pregnant women. However, these cells are known to play an important role in promoting fetal survival, and it is suggested that their increase is related to the increase in effector Th2 cells. *In vivo* and *in vitro* studies show that progesterone has the capacity to suppress Th1/Th17 differentiation and, in contrast, enhance Th2 and Treg cell differentiation. Moreover, maternal cytokines lead to greater differentiation of native T-cells into Th2 cells.^8^ The final result is an immunological imbalance, with an increase in the Th2 cell population, at the expense of Th1 and Th17 cells. It has been suggested that there is a greater targeting of Th2 cells, which is essential for the maintenance of the normal pregnancy state. It is important to emphasize that an initial environment with Th1 predominance is necessary for the success of uterine implantation. However, at the later stages of pregnancy, the predominance of the Th2 lymphocyte response is necessary for an environment of transient tolerance to fetal antigens (Fig. 1).

Considering the physiological changes in the immune response during pregnancy, the occurrence of autoimmune diseases in pregnant women is a high-risk factor and can lead to changes in their disease status during this period.^3^

Pregnancy is characterized by predominance of the Th2 lymphocyte response, a profile that coincides with the development of autoimmune bullous dermatoses. Due to the reduction in the Th1 response and possibly in Th17 during normal pregnancy, skin diseases with a predominance of the Th1 response, such as psoriasis, rheumatoid arthritis, and multiple sclerosis, show improvement during pregnancy. On the other hand, diseases with a Th2 response pattern such as pemphigus, lupus erythematosus, and asthma may not benefit from this alteration in the immune pattern, due to the predominance of the Th2 response in the activity of these diseases.^2^, ^9^

Thus, the coexistence of autoimmune bullous dermatosis in pregnant women would precipitate or increase the occurrence of pemphigus and pemphigoids (Table 1). However, the course of AIBD tends to oscillate during pregnancy due to immunological alterations caused by estrogen and progesterone levels, but also due to cortisol, norepinephrine, and dehydroepiandrosterone.^2^

**Table 1:** Course of autoimmune bullous diseases and adverse effects during pregnancy.

Disease	Course of the disease during pregnancy	Adverse effects	Neonatal bullous disease
Pemphigus vulgaris	May improve in the third trimester but recur in the postpartum period	Up to 10%, in a series of cases associated with poor disease control. Risk of premature birth in severe illness	Transient and easily managed
Pemphigus foliaceus	Variable course	Minimal	Extremely rare
Bullous pemphigoid	Rarely seen in pregnancy	Not reported	Not reported
Linear IgA Dermatosis	May improve in the second trimester but recur in the postpartum period	Minimal	Not reported
Epidermolysis bullosa acquisita	Extremely rare	A case of exacerbation during pregnancy	One case described
Gestational Pemphigoid	Tends to occur from the second to the third trimester	Increased risk of preterm birth, newborn small for gestational age or underweight	Extremely rare

Moreover, pregnancy brings other demands on the usual clinical management of AIBD: restrictions on the usual diagnostic confirmation methods, limitation in the use of certain medications, and choice of the type of delivery. For these reasons, patients with AIBD of childbearing age and who wish to become pregnant should be advised to use family planning, aiming to ensure better health conditions for her and the fetus. The use of a contraceptive method should be instituted until the disease is well controlled so that pregnancy occurs during a period with reduced disease activity.^3^, ^10^

## Some etiological and epidemiological aspects of AIBD

### Pemphigus vulgaris

There is a predominance of females in PV, demonstrated by the female: male ratio of 1.4 and 2.3, according to epidemiological studies carried out in China and Greece.^3^, ^11^ A worse outcome of PV has also been observed in women; a recent analysis showed that the HLA alleles DRB1*04:02 and DQB1 *03:02 were associated with severe PV, and DQB1*03:02 was found more frequently in female patients.

A review involving 49 pregnant women with PV found that 37% had the disease for the first time during pregnancy, and adverse pregnancy outcomes (i.e., neonatal pemphigus and perinatal death) occurred in up to 10% of cases. PV tends to improve in pregnancy, after the third trimester, and reactivate in the postpartum period. Therefore, pregnancy can precipitate or worsen PV.^12^

The occurrence of neonatal PV is transitory, with its resolution occurring within three weeks; it is usually due to transplacental transmission of IgG antibodies from mother to fetus and is easily treatable.^13^ In addition to neonatal PV, other consequences for the newborn include intrauterine growth restriction, preterm newborn, and fetal death; however, it is difficult to differentiate the consequences of treatment from those of the disease itself.^14^ Moreover, differences in desmoglein expression in neonatal skin account for the diversity of clinical manifestations between mother and child.^13^ Adverse events seem to be more correlated with poor control of maternal disease, higher serum and umbilical cord level of maternal antibodies, than with specific medications used to treat maternal PV.^15^ Therefore, treatments that lead to decreased maternal serum antibody levels aiming to limit the transplacental passage of pathogenic IgG justify the immunosuppressive therapy, although this approach during pregnancy is not well established yet.^15^ It should be remembered that, before the introduction of systemic corticosteroid use for PV, the disease had high mortality rates.

### Pemphigus foliaceus

PF has two forms: the classic (Cazenave's disease) and the endemic form. The latter includes *Fogo Selvagem* (FS), which corresponds to the South American endemic form of the disease.^16^ There is no report of gender predominance in PF or FS, but the endemic forms of PF observed in North Africa (Tunisia) show a characteristic female predominance, especially among young women (female-male ratio of 4:1). In a series of cases of 23 Tunisian patients with pemphigus, the triggering role of pregnancy in the development of this disease was suspected, as Tunisia is a country where young women have a high fertility rate.^17^

PF can have a variable course during pregnancy, with no effect on the fetus. The association with neonatal PF is extremely rare in the context of exceptionally high antibody titers.^18^ Desmoglein 3 is expressed throughout the epidermis, including the subcorneal layers in neonatal skin, and may be protective against disease-causing anti-desmoglein 1 antibodies.^13^, ^19^

A study carried out in Brazil with 19 pregnant women with FS did not observe any clinical neonatal disease, direct immunofluorescence was negative in 12 of the 17 neonatal skin specimens, IgG autoantibodies were present in low titers (less than 1:40) and IgG4 was the predominant IgG subclass in 9 of 19 umbilical cord blood samples. The authors suggest that the placenta may serve as a biological immunosorbent for pathogenic autoantibodies.^20^

### Epidermolysis bullosa acquisita

EBA is a rare, phenotypically heterogeneous subtype of AIBD, predominant in females and characterized by autoantibodies against type VII collagen. Two studies analyzed EBA and its associated data, with a total of 83 cases, showing a female-male ratio of 1.56.^21^, ^22^

### Linear IgA Dermatosis

Linear immunoglobulin A (IgA) dermatosis (LAD) is a rare autoimmune bullous disease with subepidermal blisters, in which IgA autoantibodies recognize as antigens the collagen type XVII subepidermal ectodomains (COL17, BP180) in the basement membrane zone (BMZ). There is scarce epidemiological data on its occurrence by sex, as well as its incidence in pregnancy.^23^ Patients are categorized into two groups: one with prepubertal onset and the other with a later onset (adult LAD). Some patients with LAD are young women who have become pregnant. Little is known about the effect of LAD and its treatment during pregnancy, or about the effect of pregnancy on LAD. Its antigen is known to be expressed in the amniotic basement membrane and IgA deposition occurs in the area of ​​the amniotic basement membrane of the affected mother’s fetus.^24^

According to a case series of LAD in pregnancy, the disease usually improves by the end of the first trimester and usually recurs in the second trimester but it may relapse in the postpartum period. For this reason, it may be possible to discontinue dapsone use during pregnancy. There is evidence that IgA antibodies become glycosylated during pregnancy, which could explain this remission, and Treg cells would also play a role. However, patients should be counseled about the possibility of a postpartum relapse. The disease can also reactivate years later, even after a prolonged period of remission.^25^

### Dermatitis herpetiformis

DH is closely associated with celiac disease, with both conditions being mediated by IgA autoantibodies and targeting the transglutaminase autoantigen. In contrast to pemphigus and pemphigoid, the evidence for sex predominance in DH is conflicting, with no conclusion on its prevalence in either the female or male sex.^26^ As for its occurrence during pregnancy, there is scarcity of data, with only two articles found in the literature.

## AIBD and pregnancy

The present study focused mainly on case reports and retrospective studies. The references were retrieved in a publication period going from 1979 to 2017 using an electronic search strategy: “(*Pemphigus vulgaris* [MeSH Terms]) AND (pregnancy [MeSH Terms]); (*Pemphigus foliaceus* [MeSH Terms]) E (gestation[MeSH Terms]); (*dermatitis herpetiformes* [MeSH Terms]) AND (pregnancy [MeSH Terms]); (*Epidermolysis bullosa acquisita* [MeSH Terms]) AND (pregnancy [MeSH Terms]); (*linear IgA dermatosis* [MeSH Terms]) AND (pregnancy [MeSH Terms]); Filters: case reports” on PubMed. Articles not published in English and those on gestational pemphigoid and congenital forms of bullous diseases were excluded. Fifty-six articles were included according to their titles and abstracts.

### Pemphigus and pregnancy

Forty-nine articles were included (Appendix A), which reported a total of 57 women with 59 pregnancies (Table 2); 31 patients (54.3%) were diagnosed with pemphigus before pregnancy and 26 (45.6%) during pregnancy. Fifty-one patients (89.4%) were diagnosed with *pemphigus vulgaris* and 6 (10.5%) with *pemphigus foliaceus*. The mean age at disease onset was 28.4 years and the mean gestational week at disease onset/worsening was 16 weeks. The evolution of the disease was reported in 50 pregnancies. In 33/50 (66%) pregnancies the disease worsened, in 14/50 (28%) the disease remained stable, and in 3/50 (6%) the disease improved. In twelve of the 50 pregnancies (24%), a postpartum reactivation was reported, and adverse pregnancy outcomes such as neonatal pemphigus, perinatal death, or miscarriage were reported in 33% of these pregnancies. As for the treatment of pemphigus during pregnancy, 51 patients had their treatments described. In 41 of these 51 patients (80.3%), monotherapy was the treatment of choice and in 10 of 51 (19.6%), combination therapy was reported. Rituximab was used in 4 of 51 cases (7.8%); systemic steroids in all 51 (100%); azathioprine in 5 of 51 (9.8%); mycophenolate mofetil in 3 of 51 (5.8%); dapsone in 1 of 51 (1.9%) and intravenous immunoglobulin in 4 of 51 (7.8%).

**Table 2:** Clinical and demographic data of pregnant women with pemphigus.

Authors^a^	Number of women	Diagnosis before pregnancy	Diagnosis during pregnancy	Disease	Age at onset (years)	Week of gestation at the onset or worsening	Worsening during pregnancy	Improvement during pregnancy	Stable during pregnancy	Worsening in postpartum	Newborn with bullous lesions	Treatment
Lake et al.	1	X		PV	24	NA			X			C/MPM/AZA/IVIG/RTX
	1	X		PV	19	NA			X			C/MPM/IVIG/RTX
Rangel	1		X	PV	24	11	X			X		C/RTX
Elmuradi et al.	1	X		PV	33	24	X					C/MPM/IVIG/RTX
Çayirli et al.	1	X		PV	NA	18	X					C/AZA
Salzberg et al.	1		X	PV	34	6	X					C
Kodagali et al.	1	X		PV	NA	NA					X	C
Solís-Arias et al.	1	X		PV	21	3	X			X		C
Itsukaichi et al.	1	X		PV	34	NA	X			X	X	
Ibrahim et al.	1		X	PV	23	22	X				X	C/IVIG
Lorente Lavirgen et al.	1	X		PF	33	24	X				X	C
Drenovska et al.	1		X	PV	27	NA			X	X		C
	1	X		PV	24				X			C
Galarza et al.	1		X	PF	36	17						C
Gushi et al.	1	X		PV	32	NA			X		X	C
Lehman et al.	1	X		PV	32	NA			X			
Amer et al.	1	X		PV	NA	NA			X			C
Ugajin et al.	1	X		PV	30	NA	X			X		C
Bonifazi et al.	1		X	PV	NA	NA				PV onset 6 months post- partum	X	
Fenniche et al.	1	X		PV	29	12		X		X		C
López-Jornet et al.	1		X	PV	32	20	X					C
Shieh et al.	1		X	PV	29	16	X				X	C
Okubo et al.	1		X	PV	24	18		X		X		C
Hirsch et al.	1	X		PF	23	NA					X	
Parlowsky et al.	1	X		PV	NA	NA	X			X		
Campo-Voegeli et al.	1		X	PV	35	8	X				X	C
Kalayciyan et al.	1		X	PV	31	32						C/AZA
	1		X	PV	33	8						C
	1		X	PV	32	10						C
	1		X	PV	30	4						C
Muhammad et al.	1		X	PV	28	8	X					C
Masson et al.	1	X		PV	38	12	X					C
Avalos-Díaz et al.	1	X		PF	20	NA	X				X	C
Piontek et al.	1	X		PV	29	12	X					C
Fainaru et al.	1	X		PV	32	13	X					C
Kanwar et al.	1		X	PV	33	24	X				X	C
Hern et al.	1		X	PV	32	14	X					C
	1	X		PV	33	14	X					Symptomatic treatment
Chowdhury et al.	1	X		PV	31	18			X		X	C
Virgili et al.	1		X	PV	27	26	X					C
Tope et al.	1	X		PV	27	NA		X			X	C
Goldberg et al.	1	X		PV	21	28	X					C
Kanwar et al.	1		X	PV	21	6			X			C
Moncada et al.	1	X		PV	28	16			X	X		C
Kaufman et al.	1		X	PF	27	16	X					C
Eyre et al.	1	X		PF	37	NA	X					C
Hup et al.	1	X		PV	20	NA			X			
Merlob et al.	1	X		PV	24	NA			X		X	C
Ross et al.	1		X	PV	19	13	X				Fetal death	C/AZA
Wasserstrum et al.	1	X		PV	30	20	X				Fetal death	C/AZA
Green et al.	1	X		PV	36	28	X				Fetal death	C
Moncada et al.	1		X	PV	18	14	X					C
Honeyman et al.	1		X	PV	25	32	X					C
			X	PV	29	NA	NA			X (post- miscarriage)	Miscarriage	
			X	PV	33	16	X					C
Terpstra et al.	1		X	PV	23	12	X				Fetal death	C/DAPSONE
Stanoeva et al.	1	X		PV	33	13	X					C
	1		X	PV	28	20			X	X		C
	1		X	PV	29	20			X			C

PV, Pemphigus Vulgaris; PF, Pemphigus Foliaceus; NA, data Not Available; C, Corticosteroids; MPM, Mycophenolate Mofetil; AZA, Azathioprine; RTX, Rituximab; IVIG, Intravenous Immunoglobulin.

a Bibliographic references are available as supplementary material (Appendix A).

### Linear IgA dermatosis and pregnancy

Three articles were found on LAD and pregnancy. In one of them, 12 patients had a total of 19 pregnancies. The disease remained active in 13 of 19 pregnancies, underwent remission in four of 19 pregnancies, and showed recurrence in the postpartum period in two of 19 pregnancies. The age at LAD onset ranged from 1.5 to 33 years; three patients had chronic bullous disease of childhood, and nine had adult-onset LAD. Overall, LAD tends to improve during pregnancy, especially around the 10^th^ week of pregnancy. Twelve of 19 patients received systemic treatment (7/12 received dapsone), 4/19 were in remission, and 1/19 had minimal disease without therapy.^25^

Two case report articles described LAD and pregnancy: one refers to a 24-year-old woman diagnosed with LAD and hydatidiform mole, excised in her third pregnancy, and the other refers to a 29-year-old woman in her 38^th^ week of gestation, with autoantibodies against the non-collagen 16A domain (NC16A) of COL17.^23^, ^27^

### Epidermolysis bullosa acquisita and pregnancy

Three articles on EBA and pregnancy were retrieved. In one, a 32-year-old woman was diagnosed with EBA and, at the time of delivery, both mother and newborn had superficial and deep erosions on the face, chest, abdomen, and extremities.^28^ The second article describes a patient who developed EBA on the 2nd postpartum day with resolution of the blisters at menopause.^29^ The third article reports on a 26-year-old woman who had a recurrence of EBA during the first month of pregnancy. Due to insufficient response to systemic corticosteroids, she had a miscarriage in the 2^nd^ month of pregnancy and then had a significant reduction in the number of bullous lesions.^30^

### Dermatitis herpetiformis and pregnancy

Only one article was retrieved. A 33-year-old female patient with severe DH became pregnant, requiring oral dapsone (25–50 mg per day) to control the disease. She gave birth to a full-term, healthy newborn, and progressed with disease reactivation in the postpartum period.^31^

### Laboratory diagnosis

For all cases with clinical suspicion of AIBD, the diagnosis is confirmed through cytological examination, skin biopsy with histopathology, direct and indirect immunofluorescence, and ELISA. Therefore, a patient with a confirmed diagnosis of AIBD prior to pregnancy does not need to be resubmitted to investigative workup, unless the current lesions do not show typical characteristics of the previously diagnosed AIBD. However, diagnostic confirmation is necessary in cases with symptoms suggestive of AIBD for the first time during pregnancy. Regarding the skin biopsy, topical application of chlorhexidine for antisepsis can be safely used in pregnant women. There are no reports of teratogenic effects related to topical anesthetics. However, a skin biopsy should be performed with caution in pregnant women due to the risk of fetal methemoglobinemia associated with prilocaine and decreased uterine flow secondary to the use of local anesthetics.^32^ For these reasons, alternative methods may be useful and include serological investigation using indirect immunofluorescence, ELISA, and biochip immunofluorescence microscopy.^33^

## Treatment of AIBD during pregnancy and lactation

The challenge of treating AIBDs during pregnancy refers to the choice of medication, with the main issue being the safety of the mother and the fetus. The difficulty in assessing the best and safest medications for AIBD in pregnant women is due to: 1) The rare occurrence of AIBD during pregnancy, 2) The exclusion of pregnant women from clinical drug trials, and 3) The hormonal alterations associated with pregnancy that may impact disease control by interfering with the immune response. Since the new regulations for drug use during pregnancy and lactation went into effect on June 30, 2015, the Federal Drug Administration (FDA) has eliminated the standard category letters for prescription drugs during pregnancy (A, B, C, D, and X).^34^ According to the system of the previous category, no medication for the treatment of AIBD belonged to class A, as pregnant patients could not participate in randomized controlled clinical trials.

Several drugs frequently used during pregnancy belonged to category C, as animal models showed adverse effects on the fetus. However, there are significant differences between rodents and the human placenta, and therefore these risks are often not applicable to human beings. Many of these drugs, formerly classified as category C, are in fact good choices for use during pregnancy, especially when the risks of AIBD outweigh the risks of using the drug.^10^, ^35^

### Topical glucocorticoids

Topical glucocorticoids are often the first line of treatment for AIBD and remain safe for use in pregnancy, with the advantage of not sharing the same risks as systemic steroids.^36^

Apgar scores, congenital abnormalities including orofacial clefts, preterm birth, type of delivery, and fetal death were not related to the use of topical glucocorticoids of whatever potency. One exception was the dose-response association between topical glucocorticoids and intrauterine growth restriction (IUGR) and low birth weight (LBW) newborn, specifically when applied in the third trimester at very high doses.^36^

Topical glucocorticoids are safe during lactation, as transmission through breast milk is negligible and glucocorticoids constitute a natural element of breast milk.^37^ However, high-potency topical glucocorticoids should not be applied to the nipple prior to breastfeeding, due to the risk of infant high blood pressure.^38^

### Topical calcineurin inhibitors

Case reports and a double-blind, placebo-controlled clinical trial have demonstrated the efficacy of topical calcineurin inhibitors in the treatment of AIBD, particularly if the disease is mild and localized.^39^ These agents are often used to prevent the side effects of long-term topical steroid use, especially in areas such as the face, neck, skin folds, and genitals.^10^

The effects on the fetus, which can be extrapolated from studies on the systemic administration of tacrolimus in transplant patients, include preterm delivery and LBW newborn. Although formal studies have not been carried out in pregnant women, experts recommend using a maximum of 5 g per day, for 2 to 3 weeks, in small areas.^40^

Safety recommendation data for topical tacrolimus during lactation is limited; therefore, it should be used sparingly, avoiding its use on the nipple area.^41^

### Systemic glucocorticoids

Systemic steroids are still considered an important and safe treatment option for AIBD in pregnancy. Non-fluorinated steroids, such as prednisone and hydrocortisone, are generally the preferred forms because the inactivation by 11-beta-hydroxysteroid dehydrogenase limits fetal exposure.^10^ The risks for the fetus are orofacial clefts, IUGR, LBW newborn, premature rupture of the membranes, delayed neuropsychomotor development, and preterm birth, whereas for pregnant women the main side effects include pre-eclampsia, eclampsia, high blood pressure, gestational diabetes, osteoporosis, poor wound healing, striae formation, and postpartum and postoperative infections.^10^, ^40^, ^41^ The recommended dose should not exceed 20 mg/day of prednisone, and long-term use should be avoided.^41^ If higher doses are required, the option is to switch to a steroid-sparing agent for disease control.

Maternal breastfeeding should take place at least 4 hours after prednisone administration, as its serum half-life is 60 min, with levels in breast milk peaking after 2 hours of ingestion and declining rapidly thereafter.^10^, ^40^

### Azathioprine

Azathioprine is an off-label synthetic purine-based analog used in the treatment of AIBD.^10^, ^40^, ^41^ Several clinical studies and reviews recommend azathioprine as a relatively safe immunosuppressant to be used during pregnancy and lactation.^42^ The placenta can limit the entry of azathioprine and its metabolite, 6-mercaptopurine. Transient lymphopenia and immunodeficiency can be prevented if the daily dose does not exceed 2 mg/kg/day.^43^

The European Crohn's and Colitis Organisation classifies azathioprine as “probably safe” for use during breastfeeding.^42^ Breastfeeding should take place at least 4 hours after the last dose.^44^

### Mycophenolate mofetil

Mycophenolate mofetil (MPM) is an excellent steroid-sparing agent in autoimmune diseases, including AIBD. However, it is a known teratogenic component and should not be used during pregnancy.^45^ Like methotrexate, MPM inhibits DNA synthesis and stops cell division, causing an increased risk of miscarriage. MPM has been associated with miscarriages (especially in the first trimester), limb and face abnormalities, midline defects, and other congenital malformations.^45^

Despite the scarcity of data on the use of MPM during breastfeeding, experts currently classify it as contraindicated. The small molecular weight of MPM allows it to pass in significant amounts into breast milk, which could cause developmental problems and risk of infection in the newborn.^38^

### Dapsone

Dapsone (diaminodiphenylsulfone) is often used as adjuvant therapy for AIBD. Case reports have shown it is safe to use it during pregnancy, with no evidence of teratogenicity, as well as in studies of pregnant women being treated for malaria, leprosy, or pneumonia caused by *Pneumocystis jiroveci*.^41^ Dapsone, especially at a dose > 50 mg/day, should be avoided in mothers or fetuses with glucose-6-phosphate dehydrogenase (G6PD) deficiency, due to the risk of hemolytic anemia, and all patients should be tested before use.^46^

The American Academy of Pediatrics considers dapsone to be compatible with breastfeeding, as only small amounts of its metabolites are excreted in breast milk. Jaundice in the baby should be monitored if breastfeeding occurs during dapsone therapy, especially in case of G6PD deficiency diagnosed in the newborn.

### Doxycycline

Doxycycline is an antibiotic with anti-inflammatory effects and can be useful in the treatment of AIBD.^47^ Doxycycline has long been combined with other tetracyclines and classified as unsafe during pregnancy, but this combination may not be correct, as doxycycline was developed after the toxicity of the other tetracyclines was well established. Doxycycline is likely to have a different safety profile from other tetracyclines, with no evidence to support overlapping toxicities. Tetracyclines carry an increased risk of teratogenicity, tooth discoloration, bone growth arrest, and maternal hepatotoxicity, but doxycycline does not seem to be associated with these risks. The data that differentiates doxycycline from tetracyclines is still limited, so clinicians tend to avoid doxycycline when there is a choice for other drugs with a known safety profile.^48^

Short-term doxycycline use during lactation is not contraindicated, but the drug is excreted in breast milk and studies are still scarce.^35^

### Rituximab

Rituximab (RTX) is a chimeric anti-CD20 monoclonal antibody that targets B cells, involved in the pathogenesis of AIBD. RTX is successful in providing disease remission, but it is contraindicated during pregnancy, as it crosses the placenta, depletes fetal B cells, and potentially increases the risk of maternal-fetal and newborn infections. The transplacental passage of RTX is minimal in the first trimester, moderate in the second, and higher in the third. RTX remains in serum for 3 to 6 months and is able to cross the placenta; therefore pregnancy planning is essential and patients are advised to wait 12 months after their last infusion before attempting to conceive.^10^, ^35^ In case reports of patients who accidentally received RTX during pregnancy, no definitive correlation was observed with adverse events, but data on the subject remain scarce.^49^

RTX is not recommended during lactation due to a lack of evidence on the subject.^50^

## Final considerations

Pregnancy is a period in which intense immunological and hormonal changes occur in the mother's body, in order to maintain the halogenic fetus and the stability of its own immune system. As pregnancy is characterized by the predominance of the Th2 lymphocyte response, similarly to AIBD, a worsening of these dermatoses would be expected. However, what was observed in this review is the variable course of AIBDs during pregnancy. Regarding pemphigus, PV tends to worsen, while PF does not usually suffer alterations. On the other hand, LAD shows an improvement trend. Due to the small number of studied cases, it is difficult to analyze the evolution of DH and EBA during pregnancy.

Overall, despite their rarity, the occurrence of AIBD during pregnancy is a special situation, considering the peculiarities of diagnostic confirmation by skin biopsy and the potential risks of some drugs for the conceptus. Glucocorticoids, azathioprine, and dapsone can be considered relatively safe options during pregnancy and breastfeeding, provided that their prescription and follow-up are strictly under medical supervision. On the contrary, mycophenolate mofetil, doxycycline and rituximab, despite being efficient alternatives for the treatment of AIBD, are contraindicated in pregnancy due to potential risks for the conceptus. Except for gestational pemphigoid, to the best of the authors' knowledge, there is no similar review of AIBD in pregnancy to date.

## Financial support

FUNADERSP (Fundo de Apoio à Dermatologia do Estado de São Paulo) – project n. 50-2017.

## Authors' contributions

Patrícia Penha Silveira Fagundes: Data collection; conception and design of the study; data analysis; writing the article; critical review of the content and approval of the final version.

Claudia Giuli Santi: Conception; review and approval of the final version.

Celina Wakisaka Maruta: Conception; review and approval of the final version.

Denise Miyamoto: Conception; review and approval of the final version.

Valeria Aoki: Conception and design of the study; data analysis; writing the article; critical review of the content and approval of the final version.

## Conflicts of interest

None declared.

## Footnotes

Appendix A Supplementary material related to this article can be found, in the online version, at doi: https://doi.org/10.1016/j.abd.2020.10.007.
