# Supplementary material for: Autoimmune bullous diseases in pregnancy: clinical and epidemiological characteristics and therapeutic approach
Source: An Bras Dermatol. 2021 Jul 23;96(5):581–90. doi: 10.1016/j.abd.2020.10.007 (PMC8441454; doi:10.1016/j.abd.2020.10.007)
Supplement: Supplementary file 1 [file mmc1.docx]

ABD-D-20-00239 – Supplementary Material

**Appendix A -** Supplementary reference list of studies included in the review (Table 2).

1. Lake EP, Huang YH, Aronson IK. Rituximab treatment of pemphigus in women of childbearing age: experience with two patients. J Dermatol Treat. 2017;28:751-2.

2. Rangel J. Pregnancy-Associated "Cutaneous Type" Pemphigus Vulgaris. Perm J. 2016;20:e101-2.

3. Elmuradi S, Ojeda D, Stoopler ET. Oral Pemphigus Vulgaris in Pregnancy. J Obstet Gynaecol Can. 2015;37:951-2.

4. Çayirli M, Tunca M, Akar A, Akpak YK. Favourable outcome of pregnancy in a patient with pemphigus vulgaris. J Obstet Gynaecol. 2015;35:747-8.

5. Salzberg KW, Gero MJ, Ragsdale BD. Pemphigus vulgaris in pregnancy. Cutis. 2014;94:206-9.

6. Kodagali SS, Subbarao SD, Hiremagaloor R. Pemphigus vulgaris in a neonate and his mother. Indian Pediatr. 2014;51:316-7.

7. Solis-Arias MP, Lammoglia-Ordiales L, Vega-Memije ME. [Pemphigus in pregnancy. A case report and literature review]. Ginecol Obstet Mex. 2014;82:64-9.

8. Itsukaichi M, Takakuwa K, Yamaguchi M, Serikawa T, Tanaka K, Kojima K, et al. Twins with neonatal pemphigus vulgaris born to a mother with pemphigus vulgaris: a case report. Pediatr Dermatol. 2013;30:e59-60.

9. Ibrahim SB, Yashodhara BM, Umakanth S, Kanagasabai S. Pemphigus vulgaris in a pregnant woman and her neonate. BMJ Case Rep. 2012;2012:bcr0220125850.

10. Lorente Lavirgen AI, Bernabeu-Wittel J, Dominguez-Cruz J, Conejo-Mir J. Neonatal pemphigus foliaceus. J Pediatr. 2012;161:768.

11. Drenovska K, Darlenski R, Kazandjieva J, Vassileva S. Pemphigus vulgaris and pregnancy. Skinmed. 2010;8:144-9.

12. Galarza C, Gutíerrez EL, Ramos W, Tello M, Ronceros G, Alvizuri S, et al. [Endemic pemphigus foliaceus in a pregnant woman. Report of one case]. Rev Med Chil. 2009;137:1205-8.

13. Gushi M, Yamamoto Y, Mine Y, Awazawa R, Nonaka K, Taira K, et al. Neonatal pemphigus vulgaris. J Dermatol. 2008;35:529-35.

14. Lehman JS, Mueller KK, Schraith DF. Do safe and effective treatment options exist for patients with active pemphigus vulgaris who plan conception and pregnancy?. Arch Dermatol. 2008;144:783-5.

15. Amer YB, Al Ajroush W. Pemphigus vulgaris in a neonate. Ann Saudi Med. 2007;27:453-5.

16. Ugajin T, Yahara H, Moriyama Y, Sato T, Nishioka K, Yokozeki H. Two siblings with neonatal pemphigus vulgaris associated with mild maternal disease. Br J Dermatol. 2007;157:192-4.

17. Bonifazi E, Milioto M, Trashlieva V, Ferrante MR, Mazzotta F, Coviello C. Neonatal pemphigus vulgaris passively transmitted from a clinically asymptomatic mother. J Am Acad Dermatol. 2006;55(5 Suppl):S113-4.

18. Fenniche S, Benmously R, Marrak H, Dhaoui A, Ammar FB, Mokhtar I. Neonatal pemphigus vulgaris in an infant born to a mother with pemphigus vulgaris in remission. Pediatr Dermatol. 2006;23:124-7.

19. López-Jornet P, Bermejo-Fenoll A. Gingival lesions as a first symptom of pemphigus vulgaris in pregnancy. Br Dent J. 2005;199:91-2.

20. Shieh S, Fang YV, Becker JL, Holm A, Beutner EH, Helm TN. Pemphigus, pregnancy, and plasmapheresis. Cutis. 2004;73:327-9.

21. Okubo S, Sato-Matsumura KC, Abe R, Aoyagi S, Akiyama M, Yokota K, et al. The use of ELISA to detect desmoglein antibodies in a pregnant woman and fetus. Arch Dermatol. 2003;139:1217-8.

22. Hirsch R, Anderson J, Weinberg JM, Burnstein P, Echt A, Fermin J, et al. Neonatal pemphigus foliaceus. J Am Acad Dermatol. 2003;49(2 Suppl Case Reports):S187-9.

23. Parlowsky T, Welzel J, Amagai M, Zillikens D, Wygold T. Neonatal pemphigus vulgaris: IgG4 autoantibodies to desmoglein 3 induce skin blisters in newborns. J Am Acad Dermatol. 2003;48:623-5.

24. Campo-Voegeli A, Muñiz F, Mascaró JM, García F, Casals M, Arimany JL, et al. Neonatal pemphigus vulgaris with extensive mucocutaneous lesions from a mother with oral pemphigus vulgaris. Br J Dermatol. 2002;147:801-5.

25.Kalayciyan A, Engin B, Serdaroglu S, Mat C, Aydemir EH, Kotogyan A. A retrospective analysis of patients with pemphigus vulgaris associated with pregnancy. Br J Dermatol. 2002;147:396-7.

26. Muhammad JK, Lewis MA, Crean SJ. Oral pemphigus vulgaris occurring during pregnancy. J Oral Pathol Med. 2002;31:121-4.

27. Masson P, Gaudy-Marqueste C, Es Sathi A, Tizeggaghine A, Touati K. [Neonatal pemphigus vulgaris]. Arch Pediatr. 2001;8:1136-7.

28. Avalos-Diaz E, Olague-Marchan M, Lopez-Swiderski A, Herrera-Esparza R, Diaz LA. Transplacental passage of maternal pemphigus foliaceus autoantibodies induces neonatal pemphigus. J Am Acad Dermatol. 2000;43:1130-4.

29. Piontek JO, Borberg H, Sollberg S, Krieg T, Hunzelmann N. Severe exacerbation of pemphigus vulgaris in pregnancy: successful treatment with plasma exchange. Br J Dermatol. 2000;143:455-6.

30. Fainaru O, Mashiach R, Kupferminc M, Shenhav M, Pauzner D, Lessing JB. Pemphigus vulgaris in pregnancy: a case report and review of literature. Hum Reprod. 2000;15:1195-7.

31. Kanwar AJ, Thami GP. Pemphigus vulgaris and pregnancy--a reappraisal. Aust N Z J Obstet Gynaecol. 1999;39:372-3.

32. Hern S, Vaughan Jones SA, Setterfield J, Du Peloux Menag H, Greaves MW, Rowlatt R, et al. Pemphigus vulgaris in pregnancy with favourable foetal prognosis. Clin Exp Dermatol. 1998;23:260-3.

33. Chowdhury MM, Natarajan S. Neonatal pemphigus vulgaris associated with mild oral pemphigus vulgaris in the mother during pregnancy. Br J Dermatol. 1998;139:500-3.

34. Virgili A, Corazza M, Vesce F, Garutti P, Mollica G, Califano A. Pemphigus in pregnancy. Acta Derm Venereol. 1995;75:172-3.

35. Tope WD, Kamino H, Briggaman RA, Rico MJ, Prose NS. Neonatal pemphigus vulgaris in a child born to a woman in remission. J Am Acad Dermatol. 1993;29:480-5.

36. Goldberg NS, DeFeo C, Kirshenbaum N. Pemphigus vulgaris and pregnancy: risk factors and recommendations. J Am Acad Dermatol. 1993;28(5 Pt 2):877-9.

37. Kanwar AJ, Kaur S, Abraham A, Nanda A. Pemphigus in pregnancy. Am J Obstet Gynecol. 1989;161:995-6.

38. Moncada B, Sandoval-Cruz JM, Baranda L, Garcia-Reyes J. Neonatal pemphigus. Int J Dermatol. 1989;28:123-4.

39. Kaufman AJ, Ahmed AR, Kaplan RP. Pemphigus, myasthenia gravis, and pregnancy. J Am Acad Dermatol. 1988;19(2 Pt 2):414-8.

40. Eyre RW, Stanley JR. Maternal pemphigus foliaceus with cell surface antibody bound in neonatal epidermis. Arch Dermatol. 1988;124:25-7.

41. Hup JM, Bruinsma RA, Boersma ER, de Jong MC. Neonatal pemphigus vulgaris: transplacental transmission of antibodies. Pediatr Dermatol. 1986;3:468-72.

42. Merlob P, Metzker A, Hazaz B, Rogovin H, Reisner SH. Neonatal pemphigus vulgaris. Pediatrics. 1986;78:1102-5.

43. Ross MG, Kane B, Frieder R, Gurevitch A, Hayashi R. Pemphigus in pregnancy: a reevaluation of fetal risk. Am J Obstet Gynecol. 1986;155:30-3.

44. Wasserstrum N, Laros RK Jr. Transplacental transmission of pemphigus. JAMA. 1983;249:1480-2.

45. Green D, Maize JC. Maternal pemphigus vulgaris with in vivo bound antibodies in the stillborn fetus. J Am Acad Dermatol. 1982;7:388-92.

46. Moncada B, Kettelsen S, Hernandez-Moctezuma JL, Ramirez F. Neonatal pemphigus vulgaris: role of passively transferred pemphigus antibodies. Br J Dermatol. 1982;106:465-7.

47. Honeyman JF, Eguiguren G, Pinto A, Honeyman AR, de la Parra MA, Navarrete W. Bullous dermatoses of pregnancy. Arch Dermatol. 1981;117:264-7.

48. Terpstra H, de Jong MC, Klokke AH. In vivo bound pemphigus antibodies in a stillborn infant. Passive intrauterine transfer of pemphigus vulgaris?. Arch Dermatol. 1979;115:316-9.

49. Stanoeva L, Konstantinov D, Vćkova M, Bitoljanu V. Association of pemphigus chronicus with pregnancy. Dermatologica. 1979;158:99-03.
